# (*E*)-*N*′-(5-Bromo-2-methoxy­benzyl­idene)-3-methoxy­benzohydrazide

**DOI:** 10.1107/S1600536809010435

**Published:** 2009-03-28

**Authors:** Cong-Ming Li, Hong-Yan Ban

**Affiliations:** a College of Science, Shenyang University, Shenyang 110044, People’s Republic of China; b School of Chemical Engineering, University of Science and Technology Liaoning, Anshan 114051, People’s Republic of China

## Abstract

In the title compound, C_16_H_15_BrN_2_O_3_, there are two independent mol­ecules (*A* and *B*) in the asymmetric unit. The major difference between the two mol­ecules is the dihedral angle formed by the aromatic rings [72.6 (2) and 18.8 (2)° for *A* and *B*, respectively]. The benzohydrazide groups are not planar and the mol­ecules exist in *trans* configurations with respect to the methyl­idene units. The mol­ecular packing is stabilized by two inter­molecular N—H⋯O hydrogen bonds, forming chains parallel to the *c* axis. Only the *A* mol­ecules of the asymmetric unit are held together by π–π inter­actions [centroid–centroid distance = 3.714 (3) Å].

## Related literature

For the biological activities of hydrazones, see: Zhong *et al.* (2007 ▶); Raj *et al.* (2007 ▶); Jimenez-Pulido *et al.* (2008 ▶). For related structures, see: Ban & Li (2008*a* ▶,*b* ▶); Yehye *et al.* (2008 ▶); Fun *et al.* (2008*a* ▶,*b* ▶);Yang *et al.* (2008 ▶); Ejsmont *et al.* (2008 ▶).
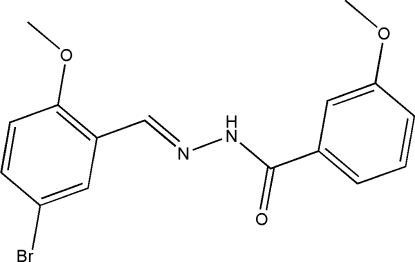


## Experimental

### 

#### Crystal data

C_16_H_15_BrN_2_O_3_
*M* *_r_* = 363.21
Monoclinic, 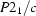

*a* = 29.063 (3) Å
*b* = 10.934 (2) Å
*c* = 9.913 (2) Å
β = 96.77 (3)°
*V* = 3128.1 (9) Å^3^
*Z* = 8
Mo *K*α radiation
μ = 2.64 mm^−1^
*T* = 298 K
0.35 × 0.33 × 0.30 mm

#### Data collection

Bruker SMART CCD area-detector diffractometer
Absorption correction: multi-scan (*SADABS*; Sheldrick, 1996 ▶) *T*_min_ = 0.458, *T*_max_ = 0.505 (expected range = 0.411–0.453)
25535 measured reflections
6785 independent reflections
3586 reflections with *I* > 2σ(*I*)
*R*_int_ = 0.076

#### Refinement

*R*[*F* ^2^ > 2σ(*F* ^2^)] = 0.058
*wR*(*F* ^2^) = 0.163
*S* = 1.03
6785 reflections
407 parameters
2 restraints
H atoms treated by a mixture of independent and constrained refinement
Δρ_max_ = 0.65 e Å^−3^
Δρ_min_ = −0.38 e Å^−3^

### 

Data collection: *SMART* (Bruker, 1998 ▶); cell refinement: *SAINT* (Bruker, 1998 ▶); data reduction: *SAINT*; program(s) used to solve structure: *SHELXS97* (Sheldrick, 2008 ▶); program(s) used to refine structure: *SHELXL97* (Sheldrick, 2008 ▶); molecular graphics: *SHELXTL* (Sheldrick, 2008 ▶); software used to prepare material for publication: *SHELXTL*.

## Supplementary Material

Crystal structure: contains datablocks I, global. DOI: 10.1107/S1600536809010435/bx2199sup1.cif

Structure factors: contains datablocks I. DOI: 10.1107/S1600536809010435/bx2199Isup2.hkl

Additional supplementary materials:  crystallographic information; 3D view; checkCIF report

## Figures and Tables

**Table 1:** Hydrogen-bond geometry (Å, °)

*D*—H⋯*A*	*D*—H	H⋯*A*	*D*⋯*A*	*D*—H⋯*A*
N2—H2⋯O2^i^	0.90 (3)	2.03 (3)	2.872 (5)	155 (5)
N4—H4*A*⋯O5^ii^	0.90 (3)	2.04 (3)	2.868 (5)	153 (5)

Symmetry codes: (i) 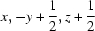; (ii) 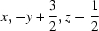.

**Fig. 1.**
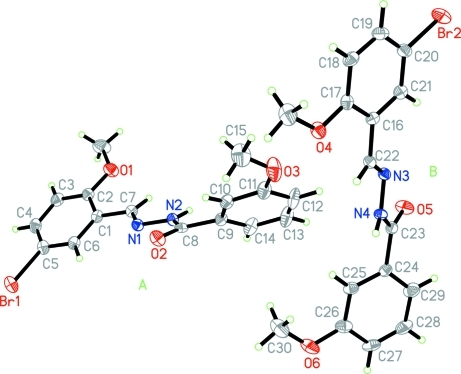
The molecular structure of (I), showing 30% probability displacement ellipsoids for the non-hydrogen atoms. The independent molecules are labelled as A and B.

**Fig. 2.**
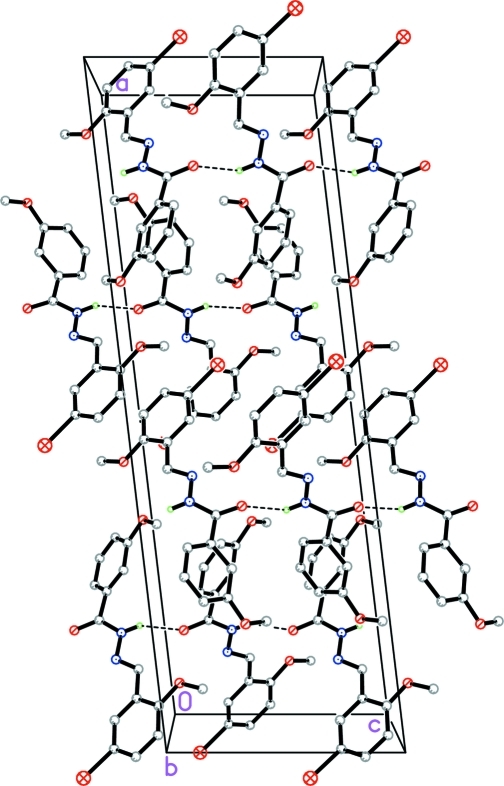
The packing diagram of (I), viewed along the b axis. Hydrogen bonds are shown as dashed lines.

## supplementary crystallographic information

### Comment

Hydrazones derived from the condensation of aldehydes with hydrazides have been demonstrated to possess excellent biological activities (Zhong *et al.*, 2007; Raj *et al.*, 2007; Jimenez-Pulido *et al.*, 2008). Due to the easy synthesis of such compounds, a great deal of hydrazones have been synthesized and structurally characterized (Yehye *et al.*, 2008; Fun *et al.*, 2008a,b; Yang *et al.*, 2008; Ejsmont *et al.*, 2008). Recently, we have reported two hydrazones (Ban & Li, 2008a,b). In this paper, we report herein the crytal structure of the title new compound, (I).

In the structure of the title compound, Fig. 1, there are two independent molecules. Each molecule exists in a *trans* configuration with respect to the methylidene unit. The dihedral angles between the two substituted benzene rings are 72.6 (2) and 18.8 (2)°, respectively. The torsion angles of C7-N1-N2-C8 and C22-N3-N4-C23 are 12.2 (3) and 4.6 (3)°, respectively.  The molecular packing is stabilized by two intermolecular N-H···O hydrogen bond to form chains parallel to the *c* axis, Fig 2. Only the A molecules of the asymmetric unit are held together by π - π interactions [Cg···Cgi(-x+1, -y,-z+1) = 3.714 (3) Å; Cg is the centroid of the C1–C6 ring].

### Experimental

The compound was prepared by refluxing 5-bromo-2-methoxybenzaldehyde (1.0 mol, 215.0 mg) with 3-methoxybenzohydrazide (1.0 mol, 166.2 mg) in methanol (100 ml). Excess methanol was removed from the mixture by distillation. The colourless solid product was filtered, and washed three times with methanol. Colourless block crystals of the title compound were obtained from a methanol solution by slow evaporation in air.

### Refinement

H2 and H4A were located in a difference Fourier map and refined isotropically, with N–H distances restrained to 0.90 (1) Å. Other H atoms were placed in calculated positions (C–H = 0.93 - 0.96 Å) and refined as riding with U_iso_(H) = 1.2U_eq_(C) and 1.5U_eq_(methyl C). A rotating group model was used for the methyl groups.

### Crystal data

**Table:**

C_16_H_15_BrN_2_O_3_	*F*(000) = 1472
*M**_r_* = 363.21	*D*_x_ = 1.542 Mg m^−^^3^
Monoclinic, *P*2_1_/*c*	Mo *K*α radiation, λ = 0.71073 Å
Hall symbol: -P 2ybc	Cell parameters from 2210 reflections
*a* = 29.063 (3) Å	θ = 2.4–25.0°
*b* = 10.934 (2) Å	µ = 2.64 mm^−^^1^
*c* = 9.913 (2) Å	*T* = 298 K
β = 96.77 (3)°	Block, colourless
*V* = 3128.1 (9) Å^3^	0.35 × 0.33 × 0.30 mm
*Z* = 8	

### Data collection

**Table:**

Bruker SMART CCD area-detector diffractometer	6785 independent reflections
Radiation source: fine-focus sealed tube	3586 reflections with *I* > 2σ(*I*)
graphite	*R*_int_ = 0.076
ω scans	θ_max_ = 27.0°, θ_min_ = 0.7°
Absorption correction: multi-scan (*SADABS*; Sheldrick, 1996)	*h* = −36→36
*T*_min_ = 0.458, *T*_max_ = 0.504	*k* = −13→13
25535 measured reflections	*l* = −12→12

### Refinement

**Table:**

Refinement on *F*^2^	Primary atom site location: structure-invariant direct methods
Least-squares matrix: full	Secondary atom site location: difference Fourier map
*R*[*F*^2^ > 2σ(*F*^2^)] = 0.058	Hydrogen site location: inferred from neighbouring sites
*wR*(*F*^2^) = 0.163	H atoms treated by a mixture of independent and constrained refinement
*S* = 1.03	*w* = 1/[σ^2^(*F*_o_^2^) + (0.0638*P*)^2^] where *P* = (*F*_o_^2^ + 2*F*_c_^2^)/3
6785 reflections	(Δ/σ)_max_ = 0.002
407 parameters	Δρ_max_ = 0.65 e Å^−^^3^
2 restraints	Δρ_min_ = −0.38 e Å^−^^3^

### Special details

**Table:**

Geometry. All esds (except the esd in the dihedral angle between two l.s. planes) are estimated using the full covariance matrix. The cell esds are taken into account individually in the estimation of esds in distances, angles and torsion angles; correlations between esds in cell parameters are only used when they are defined by crystal symmetry. An approximate (isotropic) treatment of cell esds is used for estimating esds involving l.s. planes.
Refinement. Refinement of F^2^ against ALL reflections. The weighted R-factor wR and goodness of fit S are based on F^2^, conventional R-factors R are based on F, with F set to zero for negative F^2^. The threshold expression of F^2^ > 2sigma(F^2^) is used only for calculating R-factors(gt) etc. and is not relevant to the choice of reflections for refinement. R-factors based on F^2^ are statistically about twice as large as those based on F, and R- factors based on ALL data will be even larger.

### Fractional atomic coordinates and isotropic or equivalent isotropic displacement parameters (Å^2^)

**Table:**

	*x*	*y*	*z*	*U*_iso_*/*U*_eq_	
Br1	0.438449 (17)	−0.08256 (5)	0.10000 (5)	0.0545 (2)	
Br2	1.065761 (17)	0.43703 (5)	0.40596 (5)	0.0581 (2)	
O1	0.58800 (11)	−0.0965 (3)	0.5745 (3)	0.0532 (9)	
O2	0.65071 (10)	0.2123 (3)	0.0643 (3)	0.0447 (8)	
O3	0.82081 (12)	0.3545 (4)	0.1065 (4)	0.0733 (12)	
O4	0.91879 (10)	0.3807 (3)	−0.0700 (3)	0.0421 (8)	
O5	0.85418 (11)	0.7479 (3)	0.4006 (3)	0.0490 (9)	
O6	0.68171 (11)	0.8009 (4)	0.0299 (4)	0.0617 (11)	
N1	0.60720 (11)	0.1219 (3)	0.2680 (4)	0.0322 (9)	
N2	0.64423 (12)	0.2014 (3)	0.2890 (3)	0.0311 (9)	
N3	0.89398 (11)	0.6183 (3)	0.2154 (3)	0.0294 (8)	
N4	0.85570 (11)	0.6914 (3)	0.1825 (3)	0.0279 (8)	
C1	0.55693 (15)	−0.0144 (4)	0.3641 (5)	0.0336 (11)	
C2	0.55304 (16)	−0.0983 (4)	0.4708 (5)	0.0380 (11)	
C3	0.51509 (16)	−0.1751 (4)	0.4633 (5)	0.0439 (12)	
H3	0.5126	−0.2316	0.5322	0.053*	
C4	0.48093 (16)	−0.1689 (4)	0.3547 (5)	0.0461 (13)	
H4	0.4551	−0.2194	0.3516	0.055*	
C5	0.48517 (16)	−0.0880 (4)	0.2512 (5)	0.0400 (12)	
C6	0.52270 (15)	−0.0118 (4)	0.2540 (5)	0.0371 (11)	
H6	0.5252	0.0416	0.1823	0.045*	
C7	0.59695 (15)	0.0686 (4)	0.3739 (5)	0.0346 (11)	
H7	0.6146	0.0819	0.4571	0.042*	
C8	0.66335 (14)	0.2452 (4)	0.1807 (4)	0.0295 (10)	
C9	0.70434 (15)	0.3267 (4)	0.2147 (4)	0.0341 (11)	
C10	0.74124 (15)	0.3062 (4)	0.1390 (4)	0.0386 (11)	
H10	0.7386	0.2489	0.0692	0.046*	
C11	0.78178 (17)	0.3722 (4)	0.1689 (5)	0.0487 (13)	
C12	0.7840 (2)	0.4613 (5)	0.2699 (5)	0.0573 (16)	
H12	0.8111	0.5063	0.2903	0.069*	
C13	0.7479 (2)	0.4832 (5)	0.3381 (6)	0.0625 (16)	
H13	0.7498	0.5444	0.4037	0.075*	
C14	0.70764 (19)	0.4158 (4)	0.3121 (5)	0.0522 (14)	
H14	0.6828	0.4310	0.3609	0.063*	
C15	0.82060 (19)	0.2521 (6)	0.0171 (6)	0.0752 (19)	
H15A	0.8137	0.1791	0.0647	0.113*	
H15B	0.8505	0.2443	−0.0142	0.113*	
H15C	0.7975	0.2641	−0.0592	0.113*	
C16	0.94706 (14)	0.4789 (4)	0.1332 (4)	0.0285 (10)	
C17	0.95361 (15)	0.3906 (4)	0.0343 (4)	0.0320 (10)	
C18	0.99351 (16)	0.3197 (4)	0.0470 (5)	0.0398 (12)	
H18	0.9977	0.2614	−0.0188	0.048*	
C19	1.02690 (15)	0.3349 (4)	0.1562 (5)	0.0413 (12)	
H19	1.0538	0.2880	0.1636	0.050*	
C20	1.02034 (15)	0.4200 (4)	0.2547 (5)	0.0391 (12)	
C21	0.98093 (15)	0.4919 (4)	0.2430 (4)	0.0347 (11)	
H21	0.9771	0.5497	0.3096	0.042*	
C22	0.90613 (14)	0.5574 (4)	0.1158 (4)	0.0289 (10)	
H22	0.8887	0.5630	0.0310	0.035*	
C23	0.83808 (15)	0.7528 (4)	0.2815 (5)	0.0324 (10)	
C24	0.79532 (15)	0.8269 (4)	0.2384 (4)	0.0319 (10)	
C25	0.76058 (14)	0.7787 (4)	0.1458 (4)	0.0377 (11)	
H25	0.7650	0.7046	0.1031	0.045*	
C26	0.71882 (16)	0.8424 (5)	0.1170 (5)	0.0455 (13)	
C27	0.71308 (19)	0.9548 (5)	0.1785 (6)	0.0530 (14)	
H27	0.6857	0.9986	0.1592	0.064*	
C28	0.74795 (19)	0.9991 (5)	0.2667 (6)	0.0619 (16)	
H28	0.7442	1.0747	0.3069	0.074*	
C29	0.78892 (19)	0.9369 (4)	0.2998 (5)	0.0528 (14)	
H29	0.8119	0.9692	0.3629	0.063*	
C30	0.68602 (19)	0.6876 (6)	−0.0361 (6)	0.0719 (18)	
H30A	0.7132	0.6888	−0.0826	0.108*	
H30B	0.6592	0.6741	−0.1005	0.108*	
H30C	0.6887	0.6230	0.0300	0.108*	
C31	0.92295 (18)	0.2885 (5)	−0.1714 (5)	0.0604 (16)	
H31A	0.9497	0.3053	−0.2167	0.091*	
H31B	0.8957	0.2891	−0.2363	0.091*	
H31C	0.9263	0.2097	−0.1286	0.091*	
C32	0.58764 (19)	−0.1863 (5)	0.6795 (5)	0.0688 (17)	
H32A	0.5599	−0.1774	0.7226	0.103*	
H32B	0.6142	−0.1750	0.7455	0.103*	
H32C	0.5885	−0.2666	0.6407	0.103*	
H2	0.6553 (18)	0.222 (5)	0.374 (2)	0.080*	
H4A	0.8453 (17)	0.713 (5)	0.097 (2)	0.080*	

### Atomic displacement parameters (Å^2^)

**Table:**

	*U*^11^	*U*^22^	*U*^33^	*U*^12^	*U*^13^	*U*^23^
Br1	0.0435 (3)	0.0704 (4)	0.0482 (4)	−0.0108 (3)	0.0001 (3)	−0.0070 (3)
Br2	0.0421 (3)	0.0750 (4)	0.0537 (4)	0.0093 (3)	−0.0090 (3)	0.0077 (3)
O1	0.048 (2)	0.063 (2)	0.048 (2)	−0.0067 (18)	−0.0005 (17)	0.0185 (19)
O2	0.0447 (19)	0.061 (2)	0.0287 (19)	−0.0067 (17)	0.0045 (15)	0.0080 (17)
O3	0.054 (2)	0.085 (3)	0.087 (3)	−0.033 (2)	0.035 (2)	−0.022 (3)
O4	0.0378 (19)	0.048 (2)	0.041 (2)	−0.0007 (16)	0.0059 (15)	−0.0140 (16)
O5	0.049 (2)	0.066 (2)	0.0299 (19)	0.0205 (18)	−0.0017 (16)	−0.0133 (17)
O6	0.036 (2)	0.082 (3)	0.064 (3)	0.0124 (19)	−0.0072 (18)	−0.001 (2)
N1	0.0269 (19)	0.036 (2)	0.034 (2)	−0.0021 (17)	0.0063 (17)	0.0014 (18)
N2	0.029 (2)	0.035 (2)	0.030 (2)	−0.0038 (17)	0.0073 (17)	0.0003 (18)
N3	0.0254 (19)	0.032 (2)	0.031 (2)	0.0009 (16)	0.0030 (16)	−0.0021 (17)
N4	0.0250 (19)	0.032 (2)	0.027 (2)	0.0053 (16)	0.0045 (16)	0.0020 (17)
C1	0.036 (3)	0.034 (3)	0.034 (3)	−0.002 (2)	0.013 (2)	−0.004 (2)
C2	0.036 (3)	0.037 (3)	0.042 (3)	−0.002 (2)	0.010 (2)	0.001 (2)
C3	0.047 (3)	0.037 (3)	0.050 (3)	−0.006 (2)	0.014 (3)	0.009 (2)
C4	0.040 (3)	0.043 (3)	0.057 (4)	−0.009 (2)	0.014 (3)	−0.008 (3)
C5	0.039 (3)	0.047 (3)	0.035 (3)	−0.006 (2)	0.010 (2)	−0.007 (2)
C6	0.039 (3)	0.036 (3)	0.037 (3)	−0.004 (2)	0.010 (2)	0.002 (2)
C7	0.036 (3)	0.035 (3)	0.033 (3)	0.000 (2)	0.006 (2)	0.000 (2)
C8	0.026 (2)	0.033 (3)	0.030 (3)	0.0083 (19)	0.007 (2)	0.008 (2)
C9	0.035 (3)	0.033 (3)	0.036 (3)	−0.001 (2)	0.010 (2)	0.001 (2)
C10	0.043 (3)	0.036 (3)	0.039 (3)	−0.004 (2)	0.011 (2)	0.004 (2)
C11	0.050 (3)	0.048 (3)	0.052 (3)	−0.014 (3)	0.020 (3)	0.002 (3)
C12	0.080 (4)	0.042 (3)	0.054 (4)	−0.030 (3)	0.025 (3)	−0.010 (3)
C13	0.085 (4)	0.048 (4)	0.060 (4)	−0.023 (3)	0.029 (3)	−0.019 (3)
C14	0.067 (4)	0.040 (3)	0.054 (4)	−0.009 (3)	0.028 (3)	−0.007 (3)
C15	0.053 (4)	0.095 (5)	0.086 (5)	−0.009 (3)	0.040 (3)	−0.022 (4)
C16	0.032 (2)	0.029 (2)	0.026 (2)	0.0022 (19)	0.0094 (19)	0.0029 (19)
C17	0.032 (3)	0.035 (3)	0.031 (3)	−0.006 (2)	0.010 (2)	−0.002 (2)
C18	0.044 (3)	0.032 (3)	0.046 (3)	0.005 (2)	0.017 (2)	−0.002 (2)
C19	0.034 (3)	0.040 (3)	0.051 (3)	0.009 (2)	0.010 (2)	0.013 (3)
C20	0.033 (3)	0.039 (3)	0.045 (3)	−0.001 (2)	0.001 (2)	0.015 (2)
C21	0.037 (3)	0.035 (3)	0.033 (3)	0.000 (2)	0.008 (2)	0.002 (2)
C22	0.027 (2)	0.033 (3)	0.027 (2)	−0.0007 (19)	0.0032 (19)	0.000 (2)
C23	0.036 (3)	0.029 (3)	0.032 (3)	−0.002 (2)	0.002 (2)	0.000 (2)
C24	0.039 (3)	0.029 (3)	0.028 (3)	0.002 (2)	0.006 (2)	0.002 (2)
C25	0.042 (3)	0.035 (3)	0.036 (3)	0.011 (2)	0.008 (2)	−0.002 (2)
C26	0.041 (3)	0.053 (3)	0.044 (3)	0.011 (3)	0.011 (2)	0.010 (3)
C27	0.055 (3)	0.041 (3)	0.063 (4)	0.026 (3)	0.009 (3)	0.005 (3)
C28	0.071 (4)	0.034 (3)	0.080 (4)	0.019 (3)	0.006 (3)	−0.005 (3)
C29	0.066 (4)	0.041 (3)	0.051 (3)	0.012 (3)	0.002 (3)	−0.009 (3)
C30	0.049 (3)	0.096 (5)	0.068 (4)	0.003 (3)	−0.004 (3)	−0.017 (4)
C31	0.060 (3)	0.072 (4)	0.051 (3)	−0.002 (3)	0.015 (3)	−0.031 (3)
C32	0.071 (4)	0.080 (4)	0.053 (4)	0.005 (3)	0.001 (3)	0.030 (3)

### Geometric parameters (Å, °)

**Table:**

Br1—C5	1.901 (5)	C12—H12	0.9300
Br2—C20	1.886 (5)	C13—C14	1.381 (7)
O1—C2	1.358 (5)	C13—H13	0.9300
O1—C32	1.431 (6)	C14—H14	0.9300
O2—C8	1.223 (5)	C15—H15A	0.9600
O3—C11	1.369 (5)	C15—H15B	0.9600
O3—C15	1.426 (6)	C15—H15C	0.9600
O4—C17	1.363 (5)	C16—C21	1.386 (6)
O4—C31	1.438 (5)	C16—C17	1.405 (6)
O5—C23	1.219 (5)	C16—C22	1.460 (6)
O6—C26	1.376 (6)	C17—C18	1.388 (6)
O6—C30	1.414 (6)	C18—C19	1.376 (6)
N1—C7	1.267 (5)	C18—H18	0.9300
N1—N2	1.380 (5)	C19—C20	1.378 (6)
N2—C8	1.353 (5)	C19—H19	0.9300
N2—H2	0.90 (3)	C20—C21	1.383 (6)
N3—C22	1.275 (5)	C21—H21	0.9300
N3—N4	1.377 (4)	C22—H22	0.9300
N4—C23	1.339 (5)	C23—C24	1.503 (6)
N4—H4A	0.90 (3)	C24—C29	1.371 (6)
C1—C6	1.388 (6)	C24—C25	1.386 (6)
C1—C2	1.414 (6)	C25—C26	1.399 (6)
C1—C7	1.470 (6)	C25—H25	0.9300
C2—C3	1.382 (6)	C26—C27	1.391 (7)
C3—C4	1.377 (6)	C27—C28	1.348 (7)
C3—H3	0.9300	C27—H27	0.9300
C4—C5	1.372 (6)	C28—C29	1.377 (7)
C4—H4	0.9300	C28—H28	0.9300
C5—C6	1.370 (6)	C29—H29	0.9300
C6—H6	0.9300	C30—H30A	0.9600
C7—H7	0.9300	C30—H30B	0.9600
C8—C9	1.494 (6)	C30—H30C	0.9600
C9—C14	1.367 (6)	C31—H31A	0.9600
C9—C10	1.397 (6)	C31—H31B	0.9600
C10—C11	1.384 (6)	C31—H31C	0.9600
C10—H10	0.9300	C32—H32A	0.9600
C11—C12	1.393 (7)	C32—H32B	0.9600
C12—C13	1.336 (7)	C32—H32C	0.9600
			
C2—O1—C32	118.2 (4)	C21—C16—C17	118.5 (4)
C11—O3—C15	116.7 (4)	C21—C16—C22	121.7 (4)
C17—O4—C31	118.0 (4)	C17—C16—C22	119.8 (4)
C26—O6—C30	118.2 (4)	O4—C17—C18	124.2 (4)
C7—N1—N2	114.8 (4)	O4—C17—C16	115.8 (4)
C8—N2—N1	119.3 (4)	C18—C17—C16	120.1 (4)
C8—N2—H2	122 (4)	C19—C18—C17	120.5 (4)
N1—N2—H2	119 (4)	C19—C18—H18	119.7
C22—N3—N4	114.2 (3)	C17—C18—H18	119.7
C23—N4—N3	119.1 (3)	C18—C19—C20	119.7 (4)
C23—N4—H4A	117 (3)	C18—C19—H19	120.2
N3—N4—H4A	123 (3)	C20—C19—H19	120.2
C6—C1—C2	119.2 (4)	C19—C20—C21	120.5 (4)
C6—C1—C7	121.8 (4)	C19—C20—Br2	119.2 (3)
C2—C1—C7	119.0 (4)	C21—C20—Br2	120.4 (4)
O1—C2—C3	124.8 (4)	C20—C21—C16	120.8 (4)
O1—C2—C1	116.0 (4)	C20—C21—H21	119.6
C3—C2—C1	119.2 (5)	C16—C21—H21	119.6
C4—C3—C2	120.6 (5)	N3—C22—C16	120.8 (4)
C4—C3—H3	119.7	N3—C22—H22	119.6
C2—C3—H3	119.7	C16—C22—H22	119.6
C5—C4—C3	119.8 (4)	O5—C23—N4	123.5 (4)
C5—C4—H4	120.1	O5—C23—C24	120.6 (4)
C3—C4—H4	120.1	N4—C23—C24	115.9 (4)
C6—C5—C4	121.2 (4)	C29—C24—C25	120.1 (4)
C6—C5—Br1	119.4 (4)	C29—C24—C23	119.9 (4)
C4—C5—Br1	119.4 (4)	C25—C24—C23	119.8 (4)
C5—C6—C1	119.9 (4)	C24—C25—C26	119.5 (4)
C5—C6—H6	120.0	C24—C25—H25	120.2
C1—C6—H6	120.0	C26—C25—H25	120.2
N1—C7—C1	119.4 (4)	O6—C26—C27	116.1 (4)
N1—C7—H7	120.3	O6—C26—C25	124.1 (5)
C1—C7—H7	120.3	C27—C26—C25	119.8 (5)
O2—C8—N2	122.6 (4)	C28—C27—C26	118.8 (5)
O2—C8—C9	122.1 (4)	C28—C27—H27	120.6
N2—C8—C9	115.0 (4)	C26—C27—H27	120.6
C14—C9—C10	119.9 (4)	C27—C28—C29	122.7 (5)
C14—C9—C8	124.9 (4)	C27—C28—H28	118.7
C10—C9—C8	115.2 (4)	C29—C28—H28	118.7
C11—C10—C9	119.3 (4)	C24—C29—C28	119.1 (5)
C11—C10—H10	120.4	C24—C29—H29	120.4
C9—C10—H10	120.4	C28—C29—H29	120.4
O3—C11—C10	124.0 (5)	O6—C30—H30A	109.5
O3—C11—C12	117.0 (5)	O6—C30—H30B	109.5
C10—C11—C12	119.0 (5)	H30A—C30—H30B	109.5
C13—C12—C11	121.0 (5)	O6—C30—H30C	109.5
C13—C12—H12	119.5	H30A—C30—H30C	109.5
C11—C12—H12	119.5	H30B—C30—H30C	109.5
C12—C13—C14	120.6 (5)	O4—C31—H31A	109.5
C12—C13—H13	119.7	O4—C31—H31B	109.5
C14—C13—H13	119.7	H31A—C31—H31B	109.5
C9—C14—C13	120.0 (5)	O4—C31—H31C	109.5
C9—C14—H14	120.0	H31A—C31—H31C	109.5
C13—C14—H14	120.0	H31B—C31—H31C	109.5
O3—C15—H15A	109.5	O1—C32—H32A	109.5
O3—C15—H15B	109.5	O1—C32—H32B	109.5
H15A—C15—H15B	109.5	H32A—C32—H32B	109.5
O3—C15—H15C	109.5	O1—C32—H32C	109.5
H15A—C15—H15C	109.5	H32A—C32—H32C	109.5
H15B—C15—H15C	109.5	H32B—C32—H32C	109.5
			
C7—N1—N2—C8	167.8 (4)	C12—C13—C14—C9	−0.8 (9)
C22—N3—N4—C23	175.4 (4)	C31—O4—C17—C18	1.9 (6)
C32—O1—C2—C3	4.8 (7)	C31—O4—C17—C16	−177.7 (4)
C32—O1—C2—C1	−175.0 (4)	C21—C16—C17—O4	179.0 (4)
C6—C1—C2—O1	179.4 (4)	C22—C16—C17—O4	−3.4 (6)
C7—C1—C2—O1	−1.3 (6)	C21—C16—C17—C18	−0.6 (6)
C6—C1—C2—C3	−0.4 (7)	C22—C16—C17—C18	177.0 (4)
C7—C1—C2—C3	178.9 (4)	O4—C17—C18—C19	−179.5 (4)
O1—C2—C3—C4	179.0 (4)	C16—C17—C18—C19	0.1 (7)
C1—C2—C3—C4	−1.2 (7)	C17—C18—C19—C20	0.9 (7)
C2—C3—C4—C5	1.8 (7)	C18—C19—C20—C21	−1.4 (7)
C3—C4—C5—C6	−0.7 (7)	C18—C19—C20—Br2	178.5 (3)
C3—C4—C5—Br1	178.7 (4)	C19—C20—C21—C16	0.8 (7)
C4—C5—C6—C1	−1.0 (7)	Br2—C20—C21—C16	−179.1 (3)
Br1—C5—C6—C1	179.7 (3)	C17—C16—C21—C20	0.2 (6)
C2—C1—C6—C5	1.5 (7)	C22—C16—C21—C20	−177.3 (4)
C7—C1—C6—C5	−177.8 (4)	N4—N3—C22—C16	177.8 (3)
N2—N1—C7—C1	177.6 (4)	C21—C16—C22—N3	−18.1 (6)
C6—C1—C7—N1	−18.0 (6)	C17—C16—C22—N3	164.4 (4)
C2—C1—C7—N1	162.7 (4)	N3—N4—C23—O5	0.1 (6)
N1—N2—C8—O2	−3.9 (6)	N3—N4—C23—C24	−177.8 (3)
N1—N2—C8—C9	−177.8 (3)	O5—C23—C24—C29	38.0 (6)
O2—C8—C9—C14	143.8 (5)	N4—C23—C24—C29	−144.0 (4)
N2—C8—C9—C14	−42.3 (6)	O5—C23—C24—C25	−136.1 (5)
O2—C8—C9—C10	−36.7 (6)	N4—C23—C24—C25	41.9 (6)
N2—C8—C9—C10	137.2 (4)	C29—C24—C25—C26	−0.9 (7)
C14—C9—C10—C11	3.5 (7)	C23—C24—C25—C26	173.2 (4)
C8—C9—C10—C11	−176.1 (4)	C30—O6—C26—C27	179.4 (5)
C15—O3—C11—C10	−7.5 (8)	C30—O6—C26—C25	−0.7 (7)
C15—O3—C11—C12	171.5 (5)	C24—C25—C26—O6	−178.0 (4)
C9—C10—C11—O3	176.1 (4)	C24—C25—C26—C27	1.8 (7)
C9—C10—C11—C12	−2.8 (7)	O6—C26—C27—C28	178.8 (5)
O3—C11—C12—C13	−178.7 (5)	C25—C26—C27—C28	−1.0 (8)
C10—C11—C12—C13	0.3 (8)	C26—C27—C28—C29	−0.8 (9)
C11—C12—C13—C14	1.5 (9)	C25—C24—C29—C28	−0.9 (7)
C10—C9—C14—C13	−1.7 (8)	C23—C24—C29—C28	−174.9 (5)
C8—C9—C14—C13	177.8 (5)	C27—C28—C29—C24	1.7 (9)

### Hydrogen-bond geometry (Å, °)

**Table:**

*D*—H···*A*	*D*—H	H···*A*	*D*···*A*	*D*—H···*A*
N2—H2···O2^i^	0.90 (3)	2.03 (3)	2.872 (5)	155 (5)
N4—H4A···O5^ii^	0.90 (3)	2.04 (3)	2.868 (5)	153 (5)

Symmetry codes: (i) *x*, −*y*+1/2, *z*+1/2; (ii) *x*, −*y*+3/2, *z*−1/2.
